# High-Quality Agar Polysaccharide from Unexplored *Gelidium micropterum* Kützing Biomass

**DOI:** 10.3390/polym17243278

**Published:** 2025-12-10

**Authors:** Anurag A. K. Sharma, Ravi S. Baghel, S. V. Sandhya, Rahul Kaushik, Ashok S. Jagtap, Balaji Vaishnavi

**Affiliations:** 1 Biological Oceanography Division, CSIR—National Institute of Oceanography, Panaji 403004, India; anuragaksharma1102@gmail.com (A.A.K.S.); sandhyasv.nio@csir.res.in (S.V.S.); vaishbalaji2517@gmail.com (B.V.); 2 Academy of Scientific and Innovative Research (AcSIR), Ghaziabad 201002, India; krahul.nio@csir.res.in; 3 Chemical Oceanography Division, CSIR—National Institute of Oceanography, Panaji 403004, India; 4 Arctic Ecology and Biogeochemistry, National Centre for Polar and Ocean Research, Ministry of Earth Sciences, Vasco-da-Gama 403804, India; jagtap@ncpor.res.in

**Keywords:** agar, agarophytes, alkali treatment, *Gelidium micropterum*, FT-IR, NMR

## Abstract

The agar is an important polysaccharide widely used in the food industry, pharmaceuticals, cosmetics, microbiology, and molecular biology applications. The global demand for agar polysaccharide is steadily rising, but its production is limited due to shortage of good raw material. This research investigates the potential of *Gelidium micropterum* as an alternative and sustainable source of high-quality agar. Agar was extracted using different concentrations of NaOH (4, 6, 8, and 10% *w*/*v*) and without NaOH treatment. The resulting agar yields ranged from 16.97% to 26.03%, with corresponding gel strengths between 855 ± 51 and 2078 ± 55 g/cm^2^. Notably, 8% and 10% NaOH pre-treatments yielded agar with superior gel strength and thermal properties, surpassing those reported for other Gelidiales species under similar conditions. Structural characterisation was performed using FT-IR, ^13^C-NMR, and ^1^H-NMR spectroscopy, confirming similarities with standard agar. The extracted agar’s molecular weights were in the range of 5704–7276 kDa. Sulphate content varied from 0.175 ± 0.082% to 6.197 ± 0.446% across treatments. The agar also supported microbial growth at lower concentrations than commercial agar, indicating promising application potential. These findings highlight *G. micropterum* as a promising, sustainable option for expanding agar resources.

## 1. Introduction

Red seaweeds, especially agarophytes, play a crucial role in the global hydrocolloid market. Agar, a gelatinous polysaccharide based on the disaccharide backbone of 1,3-linked-β-d-galactopyranose and 1,4-linked-3,6 anhydro-α-l-galactopyranose repeating unit. Agar extracted from red algae’s cell walls has been known for its diverse uses for a long time due to its versatile characteristics [1]. Due to its ability to gel, stabilise, or thicken a product, it is very useful in food processing, microbiological applications, pharmaceuticals, and cosmetics [2,3]. The agar market value reflects the increase in global demand, which is estimated at US$409 m in 2023 and will increase to US$629 m by 2030 with a compound annual growth rate of 6.9% (https://reports.valuates.com/market-reports/company/fujian-yange/0, accessed on 11 March 2025). The increasing global demand for agar has turned attention to exploring additional sources of agar other than the commercially known species belonging to the *Gracilaria*, *Gelidium*, and *Gelidiella* genera. Most of the agar is extracted from cultivated seaweed biomass worldwide. However, despite the abundance of red algae in Indian waters, their industrial potential remains largely underexploited, and barely 500–600 tons of dry weight of wild agarophytes are harvested on the Indian coast [4]. India continues to rely primarily on wild harvest *Gelidiella acerosa* for agar production, leaving many other species untapped [5].

India’s extensive coastline and favourable climatic conditions provide a natural habitat for diverse agarophytic seaweed species. However, the country’s agar industry has remained reliant on a narrow selection of raw materials for agar production and thus imports a huge quantity of agar to meet the national needs [6]. As global markets experience rising agar prices driven by heightened demand and limited supply, there is an urgent need to diversify the sources of agar production (https://www.chemanalyst.com/NewsAndDeals/NewsDetails/agar-agar-prices-observe-2-incline-in-asia-chemanalyst-september-data-39317, accessed on 28 October 2025). Globally, agar is commonly extracted from the members belonging to the *Gelidium* and *Gracilaria* genus [7]. Several *Gracilaria* species have been previously studied in Indian waters. Among them, two species, *Gracilaria edulis* cultivated and *Gracilaria corticata*, are growing naturally in larger quantities. However, agar extracted from these species are not suitable for microbial applications due to poor gel strength [8]. Among the Gelidiales that grow in the Indian water, *Gelidium micropterum* Kützing, found abundantly along rocky intertidal zones, thrives in challenging marine environments, forming dense clumps up to five centimetres in length [9]. Other than its wild occurrence, it was also reported as a suitable species for a land-based culture system yielding more than a 5% daily growth rate [10]. However, the suitability of this species for the extraction of agar remains unexplored.

A comparable process is normally used to prepare the commercial agar; this is performed by alkaline treatment, high-temperature extraction, filtration, and freeze–thaw cycles. This study uses the same extraction process to show industrial applicability (https://www.chemanalyst.com/Blogs/industrial-production-processes-of-agar-agar-methods-grades-and-applications-34 accessed on 3 December 2025). Certain *Gelidium* species have been previously studied, and agar yields and gel strengths have been reported with a wide range of values depending on the species and concentration of the alkali pre-treatment, namely the gel strength increasing as the alkali concentration increases [11]. As an example, *G. corneum* produced 16% native agar with a gel strength of 341 g cm^−2^ and 529 g cm^−2^ at 10% NaOH. Similarly, *G. microdon* gave 12–15% agar with gel strength up to 489 g cm^−2^, *G. sesquipedale* gave 10–12% yield with 979 g cm^−2^ gel strength, *G. pusillum* yielded 9.5–13% agar with 350–2100 g cm^−2^ strength, respectively, on the strength of alkali concentration [12,13]. Although much has been achieved on agarophytes, there is a need to explore additional seaweed species for agar extraction; *Gelidium micropterum* remains untapped for its agar extraction potential.

The concentration of sulphate in polysaccharides inhibits gelling properties, by studying previous studies, desulphation of agar polysaccharide is known to significantly improve the gel strength and rheological characteristics, as alkali treatments convert L-galactose-6-sulphate into 3,6-AG, producing good quality agar and thus diversifying its application. Thus, this study aims to explore the potential of *G. micropterum* biomass for the extraction of high-quality agar for industrial applications. For the first time, this research demonstrated the agar extraction from *G. micropterum* using various concentrations of alkaline pre-treatment. The obtained agars were characterised using FT-IR, ^13^C-NMR, and ^1^H-NMR analyses and tested for their physical properties such as gel strength, gelling, and melting temperatures. The agar’s molecular weight and sulphate content were analysed using standard methods. Lastly, the suitability of agar for the microbial culture was also investigated.

## 2. Materials and Methods

### 2.1. Sample Collection

Samples of *G. micropterum* Kützing were collected from Vagator Beach in Goa, situated on the west coast of India (15°35′56″ N, 73°43′55″ E), in October 2023. Samples were brought to the laboratory in zip-lock bags filled with seawater in an icebox. Samples were washed thoroughly with filtered seawater to remove sand, gravel, and mud. Afterwards, the barnacles and epiphytes were removed manually from the holdfasts with a brush and washed again with filtered seawater. Only morphologically identified thalli of *G. micropterum* were used for extraction, ensuring raw material homogeneity, and cleaned samples were dried at 60 °C in a hot air oven and stored in airtight zip-lock bags. The dried samples were rinsed with tap water to remove all water-soluble and insoluble components before the agar was extracted.

### 2.2. Preparation of Native Agar

Initially, 300 mL of water is used to soak 10 g of dry samples in triplicates for one hour at room temperature. Then, the seaweed was autoclaved for 1.5 h at 120 °C with distilled water (1:25, *w*/*v*). The cooked material was homogenised and then centrifuged at 6000× *g* for 5 min. The supernatant was heated again at 100 °C for 20 min and filtered through the celite bed using a vacuum filtration assembly. To obtain the agar, the supernatant was collected, kept undisturbed to form a gel at room temperature, and then frozen and thawed four times. At the end, the thawed agar was dried at 65 °C for 24 h in the hot air oven.

### 2.3. Preparation of Alkali-Treated (AT) Agar

A previously described desulphation approach was used to administer alkaline pre-treatment in the following concentrations: 4%, 6%, 8%, and 10% aqueous solutions of sodium hydroxide (NaOH) (*w*/*v*) [13]. Next, 200 mL of different concentrations of aqueous NaOH solution was added to 10 g of dried samples and incubated at 80 °C for 2 h. In order to neutralise the alkali treatment, the alkali-treated seaweed biomass was washed with tap water. This was performed until the filtrate pH reached 7–8, which is neutral or slightly alkaline. Alkali-treated material was then used to extract agar according to the above-mentioned method.

### 2.4. Testing Physical Properties

A gelatine gel tester (Parsia technology, Model: 50 N, Mumbai, India) with a cylindrical plunger that is 1 cm in diameter was used to test the extracted agar’s gel strength. For that, a 1.5% agar solution was prepared in Milli-Q water with all the extracted agar concentrations and commercial agar (SRL, Mumbai, India), which was used as a reference, and left at 10 °C overnight before determining the gel strength. The strength of the gel was determined by the weight needed to break it [14,15]. Agars’ gelling and melting temperatures were determined using the methodology described by Shukla et al. (2011) [16]. In order to determine the gelling temperature, a glass test tube containing 25 mL of 1.5% (*w*/*v*) heated agar solution was left to cool. After that, the temperature was gradually lowered and, until gelation happened, this process was monitored every minute. In order to determine the melting temperature, 1.5% (*w*/*v*) agar gel was heated with a temperature rise of roughly 0.5 °C min^−1^. The melting temperature was determined by measuring the temperature at which a glass bead with a weight of 4.93 g and a diameter of 15.78 mm sank when placed on top of the gel [11,14,17].

### 2.5. Sulphate Content Analysis

Sulphate content analysis was performed according to Torres et al. (2021) [18], 10 mg of the extracted agar sample was added in 2.5 mL of 0.5 M HCl and incubated at 105 °C for 3 h and then centrifuged at 13,400 rpm for 15 min. After centrifugation, 200 μL of the sample was taken and mixed with 1400 μL of 0.5 M HCl, vortexed, and the 1st reading was taken at 405 nm. After this, 400 μL of barium chloride–gelatine reagent was added and a 2nd reading was taken at 405 nm. The sodium sulphate was used as a reference [19].

### 2.6. FTIR Characterisation

The agar samples were finely ground for further characterisation. Fourier transform infrared (FT-IR) spectroscopy (PerkinElmer Spectrum IR Version 10.7.2, Shelton, CT, USA) was used to characterise the agar sample. A total of 16 scans of FT-IR (4000–400 cm^−1^) were captured. Agar bands were compared with the agar spectra obtained from the standard agar (SRL, Mumbai, India) as well as the agar spectra reported in the literature [20].

### 2.7. ^1^H and ^13^C-NMR Characterisation

To confirm the agar molecule, ^1^H-NMR and ^13^C–NMR spectra were recorded in NMR spectrometer with operating frequencies of 500 MHz and 125 MHz, respectively. Agar extracted from the 8% and 10% alkali pre-treatment samples in powdered form was dissolved in DMSO-d_6_ for the data acquisition. Furthermore, NMR data were recorded for the native agar and the standard agar for comparison under the same conditions and solvent.

### 2.8. Molecular Weight Determination

The molecular weight of the native agar and AT agar samples was determined by gel permeation chromatography on a PL aquagel-OH 50 column (Agilent, Santa Clara, CA, USA) using HPLC grade water as eluent at room temperature and 0.8 mL/min flow rate using a refractive index detector. A molecular weight calibration curve was drawn using Pullulan standards (9900, 50,100, 110,000, 224,000, 356,000, and 894,000 Da) (Figure S1). The samples were also determined in their molecular weight using a Pullulan standard calibration.

### 2.9. Testing Suitability of Agars for Bacteriological Application

The Tryptone Soya Broth (Hi-Media, Mumbai, India), consisting of different agars extracted in this study and commercial agar, were prepared. To create a homogeneous gel texture in all of the microbial culture plates, the concentration of agar was normalised with respect to the gel strength of the commercial agar (825 g/cm^2^ obtained with 2% *w*/*v*). Based on this, the equivalent strength of the gels has been achieved by optimisation of the alkali-treated agar (ATA) concentrations: 1.47% for 4% ATA, 0.94% for 6% ATA, 0.85% for 8% ATA, and 0.79% for 10% ATA, and 1.93% for native agar. An overnight grown culture of *Escherichia coli* was streaked on sterile Tryptone Soya Agar (TSA) plates, and growth was monitored after incubation at 37 °C for 24 h. In addition, appropriate dilution of *E. coli* culture was spread plated on sterile Tryptone Soya Agar (TSA) plates, and total bacterial count (CFU mL^−1^) on each plate was determined after incubation at 37 °C for 24 h.

## 3. Results and Discussion

### 3.1. Agar Yields

Agar was extracted, and a gel was formed, both with and without NaOH pre-treatments. On dry weights (dw), the agar yields varied from 16.97% to 26.03% (Table 1). As the concentration of NaOH pre-treatment increased, the agar yields dropped. The lowest yield was recorded for the 10% ATA, and the highest for the native agar. The breakdown of a specific percentage of polysaccharides in an alkaline solution could be the cause of this [21]. The agar yields obtained in this study were higher than the agar yields reported for other members of the family *Gelidiellaceae* and *Pterocladiaceae* (Table 2), for example *Gelidium corneum* (10–16%) *and G. microdon* (12–15%) [12]; *G. sesquipedale* (10–12%) [22]; *G. pusillum* (8–13%) [13]; and *G. pusillum* (10–19%) [8], and comparable to the yields reported from *Gelidiella acerosa* 11–28% [8], *G. floridanum* (24–32%), and *G. serrulatum* (15–33%) [23] on a respective alkaline pre-treatment concentration basis. The agar yields were also in the range that was reported for various *Gracilaria* species (Table S1) [8,24,25,26,27,28,29,30,31,32,33,34,35,36,37].

### 3.2. Agar Physical Properties

The agar (10% ATA) that was extracted with a 10% NaOH pre-treatment had the highest gel strength, followed by 8%, 6%, and 4%, and the lowest was recorded for the native agar (Table 1). Treating seaweed with NaOH (alkali) significantly enhances its gel strength by reducing sulphate content [25]. The gel strength (2078 ± 55 g/cm^2^) of *G. micropterum* agar achieved with 10% NaOH pre-treatment was higher than those agars prepared from other *Gelidium* species with similar concentrations of NaOH pre-treatment—*G. corneum* (528.55 g/cm^2^), *G. microdon* (489 g/cm^2^), *G. sesquipedale* (979 g/cm^2^), and *G. pusillum* (1500 g/cm^2^)—and comparable to G. *pusillum* (2100 g/cm^2^) and *Gelidiella acerosa* (2000 g/cm^2^) (Table 2) [12,13,14,15,16]. The agars from other agarophytic seaweeds such as *Gracilaria edulis* (490 g/cm^2^), *G. acerosa* (950 g/cm^2^), *G. corticata* (110 g/cm^2^), *G. foliifera* (135 g/cm^2^), *G. salicornia* (510 g/cm^2^), and *Phycocalidia Vietnamensis* (597 g/cm^2^) harvested from the coast of India also showed lower gel strength in comparison to *G. micropterum* agar (Table S1) [8,24,25,26,27,28,29,30,31,32,33,34,35,36,37].

The agar gelling temperatures for native agar and NaOH pre-treated agar were recorded in the range of 39.8 ± 0.25–42 ± 0.55 °C, as shown in Table 1. These temperatures were equivalent to the gelling temperature of *Gelidiella acerosa* (41 °C) and higher compared to the gelling temperature of *G. robustum* (35.3 °C) and *G. pusillum* (34 °C). The melting temperatures for the agars ranged from 90.33 ± 0.57 to 96.5 ± 0.5, respectively (Table 1), which were lower than the gelling temperatures for the agars that had been previously reported from *G. pusillum* (98 ± 1.0 °C) [31].

### 3.3. Sulphate Content

The sulphate content for the native and alkali pre-treated agars was in the range of 0.175% ± 0.082 to 6.197% ± 0.446. An increase in NaOH concentration gradually reduces the sulphate content of the agar samples [38]. Native agar showed the maximum sulphate content of 6.197% ± 0.446, followed by 4% ATA having sulphate content of 0.402% ± 0.069, 6% ATA having sulphate content of 0.2845% ± 0.021, 8% ATA having sulphate content of 0.225 ± 0.084, and the lowest sulphate content of 0.175 ± 0.082 was detected in 10% ATA. Commercial agar was taken as a reference and the sulphate content was detected to be 0.242%. The sulphate content of *G. micropterum* at 10% ATA was less than comparable to the sulphate content of 10% treated *G. pusillum* (0.32%) [13] and *Gelidiella acerosa* (0.60%) [8]. The alkali treatments were found to be effective in removing sulphate content and improving the agar quality.

### 3.4. FT-IR Analysis

**Figure 1:**
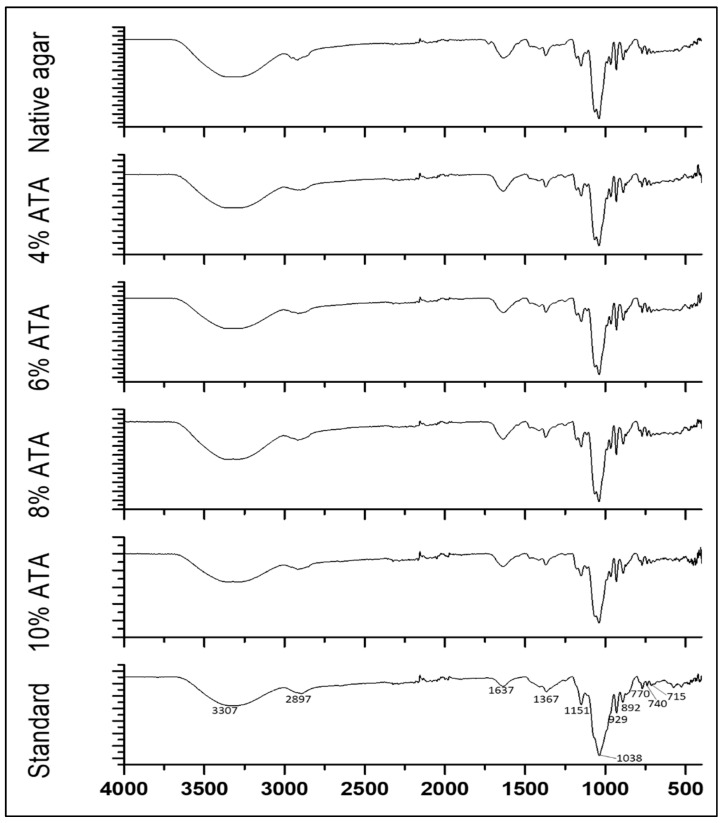
FT-IR spectra of agars obtained from *G. micropterum* biomass with different concentrations of alkali pre-treatments and standard agar.

Through distinctive absorption bands, FT-IR analysis of agar samples exposed important structural elements and functional groups. A broad band for O-H stretching in the region of 3307 cm^−1^ and peaks for methoxyl groups at 2897 cm^−1^ were recorded for all agar samples, which were in accordance with FT-IR spectra reported for the *G. pusillum* and *G. elegans* agars [13,39]. A band at 1637 cm^−1^ was recorded representing C=O and N-H groups, similarly to previous reports [13,39]. Further, all agars exhibit identical FT-IR spectra in the 500–1500 cm^−1^ range (Figure 1). The peaks at 1367 cm^−1^ correspond to the sulphate ester group comparable to those reported for *G. pusilum* [31] and *G. elegans* [39]. The strong peak at 1038 was recorded, which indicates the presence of 3,6-anhydrogalactose bridges similar to agar reported for *G. elegans* [39] and *Phycocalidia Vietnamensis* agar [2]. The distinctive bands at 715, 770, and 927 cm^−1^ are attributed to the β-galactose skeletal bending [2,13,31]. The FT-IR band at 892 cm^−1^, specific to agar, is caused by the anomeric C-H of galactose residues [31]. Additional distinguishing bands were also observed, similar to standard agar and to those reported in the previously published literature.

### 3.5. ^1^H and ^13^C-NMR Analysis

**Figure 2:**
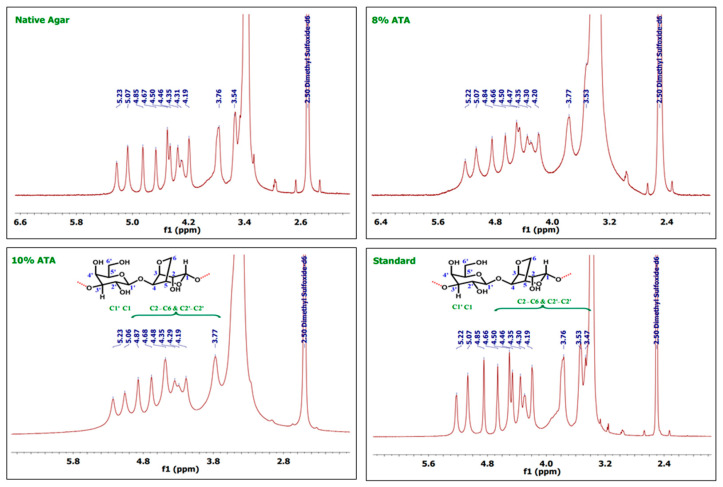
1H-NMR spectra of the agar extracted from *G. micropterum* and standard agar.

**Figure 3:**
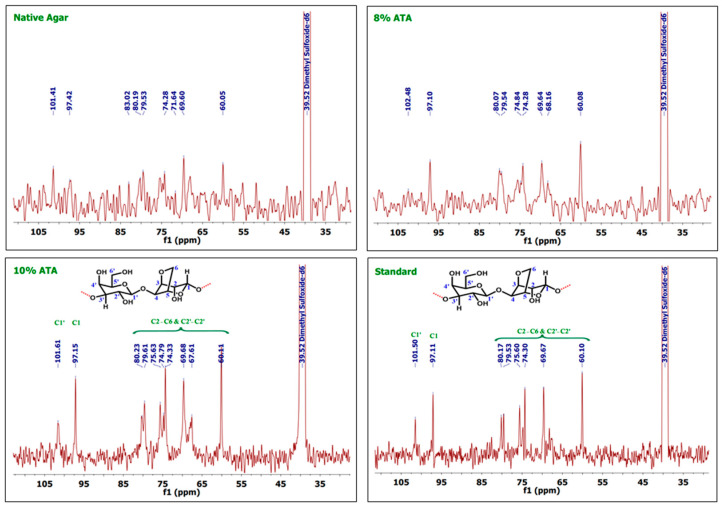
^13^C-NMR spectra of the agar extracted from *G. micropterum* and standard agar.

As discussed in the earlier reports, the ^1^H NMR shows peak for C1 protons at ppm 5.07 and C1′ protons at ppm 5.21, whereas agarose skeleton protons of (C2–C6) and (C2′–C6′) show multiplet in the region 4.84–3.8 ppm as reported by Kunihiko Izumi [40] and Trivedi and Kumar [41]. In the case of 1H-NMR, 8% ATA samples show peaks at 5.22 and 5.07, which can be attributed to the signal from the Hydrogen atoms on C1′ and C1, respectively (Figure 2). Whereas multiple and broad peaks in the region 4.84–3.77 corresponds to hydrogen atoms attached to C2–C6 and C2′–C6′, which support the formation of agar. As documented in the earlier reports, the agar skeleton contains galactose C1–C6, which shows characteristics signals at the region of 102, 70, 82, 68, 75, and 61 ppm [42,43]. In the case of 13C-NMR spectrum, carbon atoms at C1′ and C1 give signals at 102.48 and 97.10 ppm, respectively (Figure 3). The other signals at 80.07, 79.54, 74.84, 69.64, 68.16, and 60.08 can be attributed to C2–C6 and C2′–C6′ numbered carbon atoms. Similarly, for 10% ATA, peaks at 5.23 and 5.06 corresponds to C1′ and C1 hydrogen atoms, respectively, and broad peaks from 4.87 to 3.77 confirmed the presence of C2–C6 and C2′–C6′ hydrogen atoms. In 13C-NMR spectrum also, the peaks at 101.61 and 97.15 show the presence of C1′ and C1 carbon atoms. The chemical shift obtained matches the already reported data and confirms the agar structure. Whereas 80.23, 75.63, 74.79, 69.68, 67.61, and 60.11 can be attributed to C2–C6 and C2′–C6′ carbon atoms. These (1H-NMR and 13C-NMR) spectra were compared with the control and standard agar purchased from commercial sources and the chemical shift matches with the agar samples obtained in this work. Both the control sample and standard agar have shown similar peaks without any significant chemical shift, which confirms the high purity of agar obtained from 8% and 10% alkali pre-treatments.

### 3.6. Molecular Weights

**Figure 4:**
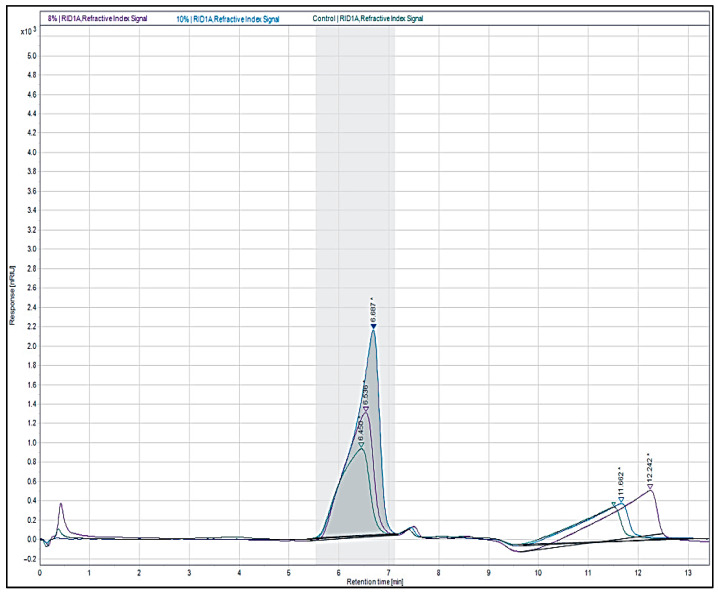
Gel permeation chromatography elution profile of native agar, 8% ATA, and 10% ATA (The asterisk represents the (*) processing of the sample to adjust the peak area, and the arrow (∇) represents the retention time of the samples).

The molecular weights for the native agar, 8% ATA and 10% ATA, were 7276.42 kDa, 6355.62 kDa, and 5704.42 kDa, respectively (Figure 4). The alkali treatment was found to reduce the agar molecular weight significantly by about 12.7% for 8% ATA and about 21.5% for 10% ATA over the native agar. The reduction in the agar molecule size with alkali treatment is because NaOH not only decreases polymer chain length but also removes sulphate content, making shorter molecules and lowering overall molecular weight in accordance with the previous report in the literature [17,44].

### 3.7. Suitability of Agars for Bacteriological Application

**Figure 5:**
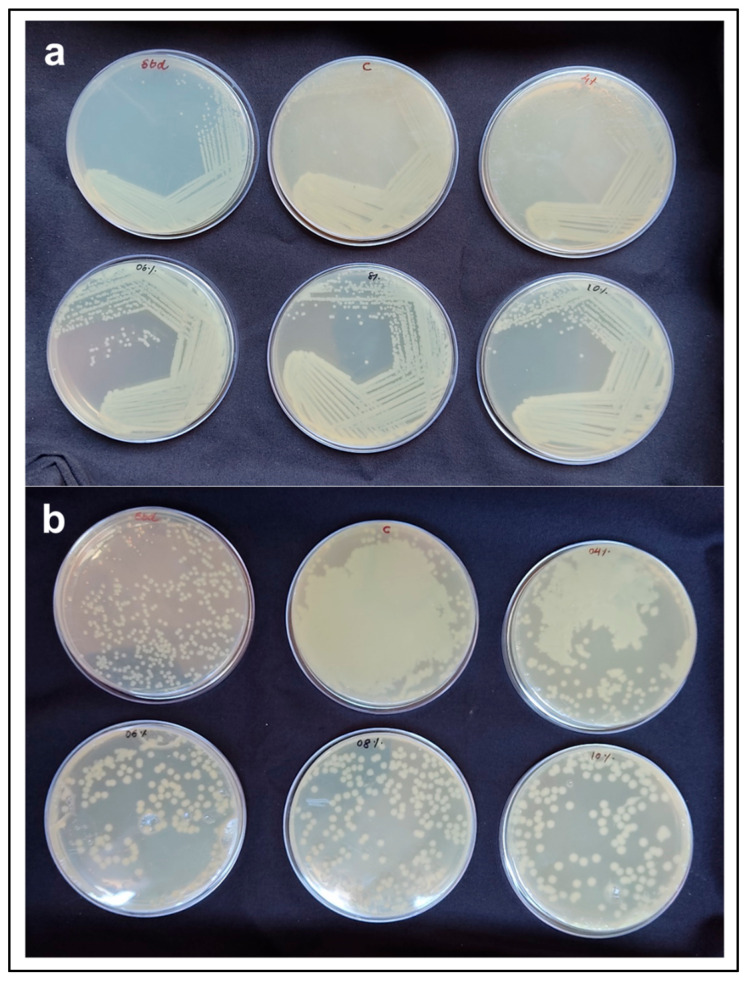
*E. coli* growth on TCA plates made with commercial agar (std) and agar extracted from *G. micropterum* biomass using different concentrations of NaOH pre-treatment (native agar (labelled as C), 4% ATA, 6% ATA, 8% ATA, and 10% ATA). (**a**) Streak plate method; (**b**) Spread plate method.

There was no difference in the colony characteristics of *E. coli* when streaked on the TSA plates made with native, alkali pre-treatment and commercial agar (Figure 5a). In the spread plating method, there was mat growth on the TSA plates with native agar and 4% ATA. However, TSA plates with 6% ATA, 8% ATA, and 10% ATA allowed distinct colony formation and measurable growth. Total count of *E. coli* on TSA plates with 6% ATA, 8% ATA, and 10% ATA were in the range of 10^8^ CFU mL^−1^ (Table 3) and *E. coli* colony count was comparable with standard agar (Figure 5b). Agar is one of the most widely used gelling agents for solidifying bacteriological media [45]. In our study, the growth characteristics of the test organisms were comparable to those observed with commercial agar, and no inhibitory effects were detected when agar extracted from *G. micropterum* biomass was used as the solidifying agent. Unlike the food industry, the microbiology field prefers agar with higher gel strengths > 700 g/cm^2^ [22]. Owing to its superior gel strength, agar extracted from *G. micropterum* can be utilised at lower concentrations for microbiological media preparation. Our results align with the findings of Reddy et al. (2018) [46], demonstrating the suitability of seaweed-extracted agar for microbial culture media preparation at lower concentrations (0.75–1.5%), comparable to commercial agar. Overall, our results clearly demonstrate that the extracted agar possesses the essential properties required for a bacteriological solidifying agent, including (i) gelling temperature below 45 °C; (ii) ability to form a firm surface for streaking without surface fissuring; (iii) minimal syneresis; (iv) no inhibitory effect on bacterial growth; and (v) economic feasibility with respect to low weight-to-volume concentration [47].

### 3.8. Applicability of Extracted Agar for Food Industry

The gel strength, melting temperature, and gelling temperature are the key defining factors of extracted agar in the food industry because they dictate the performance of agar in different applications. The food-grade agar has a gel strength of 400–1500 g/cm^2^, which provides the solidity and stability of the product that is required for food items [48]. According to the Food and Agriculture Organization, the melting temperature of food-grade agar generally ranges between 80 °C and 98 °C, while the gelling temperature is notably lower—typically between 32 °C and 45 °C. In this study, the agar extracted from *G. micropterum* is performing outstandingly even with a lower concentration of agar in every aspect (Table 1). This significant hysteresis interval ensures that agar-based gels remain stable across a broad temperature range, which is essential for products requiring heat resistance or extended shelf life. The hysteresis also allows agar gels to withstand room temperatures without melting, making them beneficial for desserts.

In practice, the capacity of agar to develop a clear, stable, and reversible gel depends on the basis of its extensive use in the food industry. It can be used as a gelling agent in jellies, puddings, and custards, a stabiliser in yoghurts, mousse, and ice creams, and a thickener in jam and confectionery. Vegetarian origin, excellent gel clarity, and thermal hysteresis makes agar a valuable gelatine substitute as well as a flexible additive in a wide range of formulations [49].

## 4. Conclusions

This study is the first to comprehensively evaluate *G. micropterum* biomass as a viable and sustainable source of high-quality agar. The species demonstrated high extraction yields and good gel strength, particularly following alkali pre-treatment. Agar extracted using 8% and 10% NaOH exhibited superior physical and thermal properties, like gel strength up to 2078 g/cm^2^, high melting temperatures, and reduced sulphate content surpassing many commercially important Gelidiales species. Structural characterisation (FT-IR, ^1^H-NMR, and ^13^C-NMR) confirmed that the extracted agar is chemically comparable to commercial-grade agar and agar structure for various seaweeds reported in the literature, while molecular weight analysis revealed that high concentrations (8 and 10%) of alkali pre-treatment significantly reduce the agar molecular weights and sulphate content.

The 10% NaOH pre-treatment produced agar with minimal sulphate content (0.175%) and a high melting temperature (96.5 °C), indicating improved gel quality and thermal stability. The extracted agar also supported microbial growth comparable to commercial agar, confirming its suitability for food, pharmaceutical, and microbiological applications. The physical properties of agars were comparable to commercial agar. Among the extracted agars, 6% ATA, 8% ATA, and 10% ATA prepared agar from *G. micropterum* could potentially be used in microbial culture applications, while 4% ATA and native agar could be used in food, pharma, cosmetics, and microbiology applications. Future studies could be focused on the cultivation of this commercially important seaweed species and on replacing alkali pre-treatments with a green process to remove the sulphate content.

## Figures and Tables

**Table 1:** Physical properties of agars obtained from *G. micropterum* biomass with different concentration of alkali pre-treatments and native agar.

NaOH Conc. % (*w*/*v*)	Agar Yields (% DW)	Gel Strength (g/cm^2^)	Gelling Temp. (°C)	Melting Temp. (°C)	Viscosity (mPa·S)
Native agar (0)	26.03 ± 1.86	855 ± 51	39.8 ± 0.25	90.33 ± 0.57	18.34 ± 2.81
4	24.1 ± 0.8	1110 ± 34	40.6 ± 0.15	91.5 ± 0.5	26.15 ± 6.41
6	20.5. ± 1.5	1762 ± 37	41 ± 0.25	94 ± 0.5	30.32 ± 5.87
8	18.06 ± 1.3	1940 ± 12	41.6 ± 0.2	94.83 ± 0.28	62.54 ± 13.61
10	16.97 ± 0.78	2078 ± 55	42 ± 0.55	96.5 ± 0.5	63.03 ± 10.66

**Table 2:** Comparison of agar yields and gel strength of agar extracted from *G. micropterum* biomass in the present study with data reported for the members of *Gelidiellaceae* and *Pterocladiaceae* family.

S. No.	Species	Alkali Pre-Treatment Conc. % (Agar Yields % and Gel Strength g/cm^2^)	References
1	*Gelidium micropterum*	0 (26 and 855), 4 (24 and 1110), 6 (20.5 and 1762), 8 (18 and 1940) 10 (16.97 and 2078)	**Present study**
2	*Gelidium corneum*	0 (16 and 341), 10 (6 and 529)	[12]
3	*Gelidium microdon*	0 (12 and 350), 10 (15 and 489)	
4	*Gelidium sesquipedale*	0 (12 and 245), 10 (3 and 979)	[22]
5	*Gelidium pusillum*	0 (13 and 350), 5 (11 and 1550), 8 (10 and 1800), 10 (9.5 and 2100), 15 (8 and 2100)	[13]
6	*Gelidiella acerosa*	0 (28 and 800), 4 (24 and 1025), 6 (18 and 1400), 8 (13 and 1850), 10 (12 and 2000), 15 (11 and 2000)	[31]
7	*Gelidium pusillum*	0 (19 and 740), 4 (18 and 1000), 6 (17 and 1200), 8 (12 and 1400), 10 (12 and 1500), 15 (10 and 1500)	
8	*Pterocladia capillacea*	0 (32.1 and 753.3), 4 (27.6 and 1470)	[23]
9	*Gelidium floridanum*	0 (31.7 and 1030), 4 (24.2 and 730)	
10	*Gelidium serrulatum*	0 (33 and 380), 4 (15.4 and 687)	

**Table 3:** Total count of *E. coli* on TSA plates with seaweed agar pre-treated with different concentrations of alkali treatment.

SI No.	Agar Type (Used Conc. %)	Total Bacterial Count (CFU mL^−1^)
1	Native agar (1.93)	Mat growth
2	4% ATA (1.47)	Mat growth
3	6% ATA (0.94)	1.89 × 10^8^
4	8% ATA (0.85)	2.32 × 10^8^
5	10% ATA (0.79)	1.4 × 10^8^
6	Commercial standard agar	4.28 × 10^8^

## Data Availability

The data supporting this article have been included as part of the Supplementary Materials.

## Supplementary Materials

The following supporting information can be downloaded at: https://www.mdpi.com/article/10.3390/polym17243278/s1, Figure S1: Pullulan standards (9900, 50,100, 110,000, 224,000, 356,000, and 894,000 Da) chromatograms; Table S1: Comparison of agar yields and gel strength of agar extracted from *G. micropterum* biomass in the present study with data reported for the members of *Gracilariaceae* and *Bangiaceae* family.

## Abbreviations

The following abbreviations are used in this manuscript:

AT	Alkali-treated
ATA	Alkali-treated Agar
DW	Dry weight
FTIR	Fourier transform infrared spectroscopy
NMR	Nuclear Magnetic Resonance
