# Supplementary material for: High-Quality Agar Polysaccharide from Unexplored Gelidium micropterum Kützing Biomass
Source: Polymers (Basel). 2025 Dec 10;17(24):3278. doi: 10.3390/polym17243278 (PMC12737070; doi:10.3390/polym17243278)
Supplement: Supplementary file 1 [file polymers-17-03278-s001.zip › polymers-3948820-supplementary.pdf]

Supplementary Material

High-quality agar polysaccharide from unexplored *Gelidium micropterum* Kützing biomass

Anurag A. K. Sharma [1,2], Ravi S. Baghel [1,2,*], S V. Sandhya [1,2], Rahul Kaushik [2,3], Ashok S. Jagtap [4] and Balaji Vaishnavi [1]

[1] Biological Oceanography Division, CSIR—National Institute of Oceanography, Panaji 403004, India; anuragaksharma1102@gmail.com (A.A.K.S.); sandhyasv.nio@csir.res.in (S.V.S.); vaishbalaji2517@gmail.com (B.V.)

[2] Academy of Scientific and Innovative Research (AcSIR), Ghaziabad 201002, India; krahul.nio@csir.res.in

[3] Chemical Oceanography Division, CSIR—National Institute of Oceanography, Panaji 403004, India

[4] Arctic Ecology and Biogeochemistry, National Centre for Polar and Ocean Research, Ministry of Earth Sciences, Vasco-da-Gama 403804, India; jagtap@ncpor.res.in

Corresponding author

Email: rsbaghel@nio.res.in; ravisingh501@gmail.com

Phone: +91 8322450295

Fax: +91 8322450606

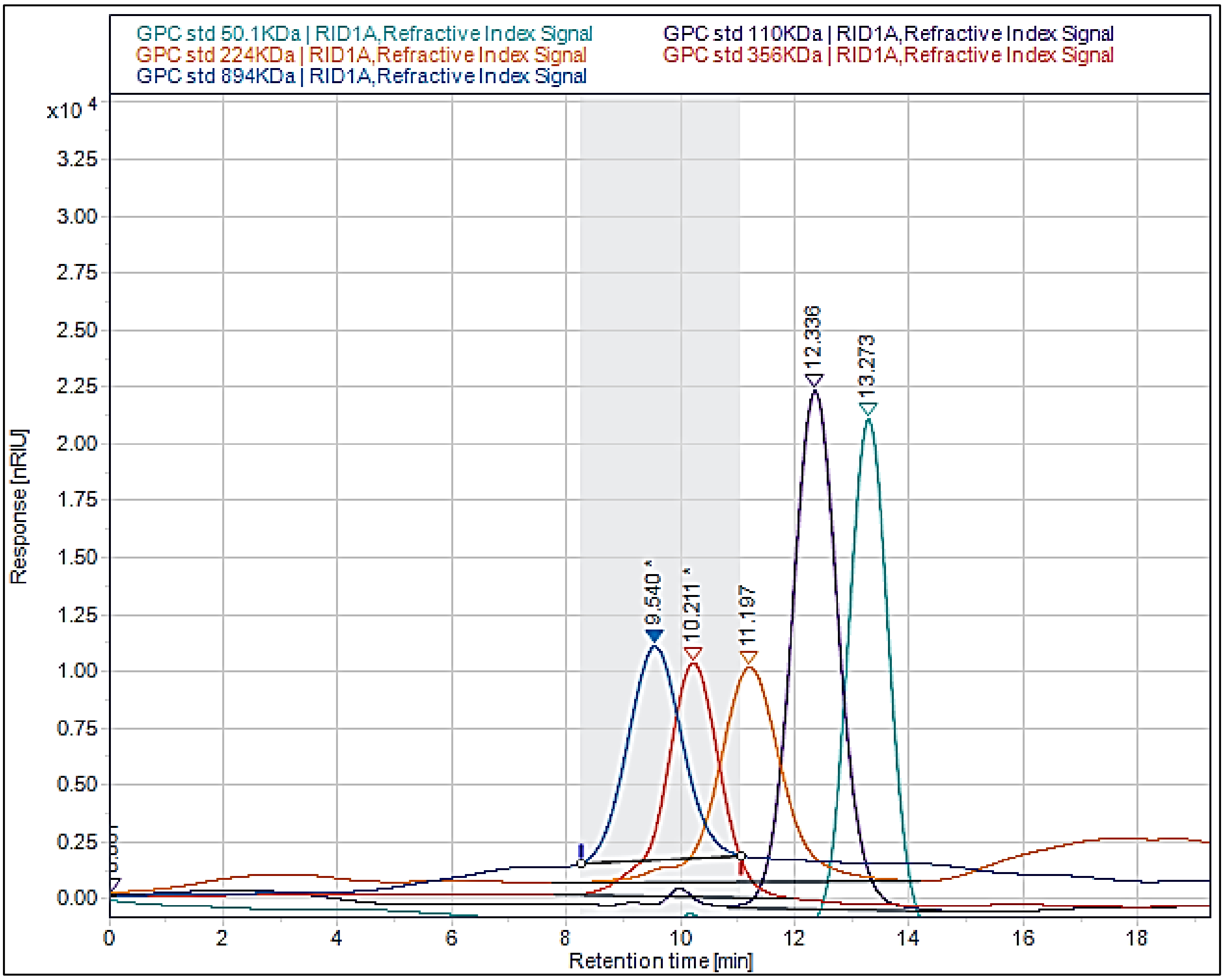

Supplementary Figure S1. Pullulan standards (9900, 50,100, 110,000, 224,000, 356,000 and 894,000 Da) chromatograms. (The asterisk represents the (*) processing of the sample to adjust the peak area, and the arrow (∇) represents the retention time of the samples.)

Supplementary Table S1 Comparison of agar yields and gel strength of agar extracted from *G. micropterum* biomass in the present study with data reported for the members of *Gracilariaceae* and *Bangiaceae* family.

**S. No.**	**Species name**	**Alkali Pre-treatment conc. % (agar yields % & gel strength g/cm²)**	**References**
11	*Gracilaria lemaneiformis*	0 (38 & 301), 7 (5 & 760)	[25]
12	*Gracilaria verrucosa*	0 (11 & 15), 1 (30 & 565), 3 (34 & 627)	[26]
13	*Gracilaria tenuistipitata*	2 (22 & 624), 4 (21 & 763), 6 (19 & 996), 8 (17 & 1199), 10 (16 & 1251)	[21]
14	*Gracilaria debilis*	8 (25-35 & 300-800)	[27]
15	*Gracilaria edulis*	8 (25 & 300-700)	
16	*Gracilaria vermiculophylla*	6 (14.4 & 1331)	[28]
17	*Gracilaria vermiculophylla*	0 (17 & 1132), 7 (28 & 170)	[29]
18	*Gracilaria vermiculophylla*	0 (15 & 115), 2 (24 & 400), 4 (28 & 500), 6 (30 & 679), 8 (27 & 500)	[30]
19	*Gracilaria lemaneiformis*	5 (17.8 & 954)	[31]
20	*Gracilaria edulis*	0 (25 &100), 3 (23 & 135), 4 (20 & 220), 6 (18 &340), 8 (16 & 490), 10 (13 & 490), 15 (11 & 490)	[23]
21	*Gracilaria crassa*	0 (23 &250), 3 (22 & 330), 4 (21 & 420), 6 (18 &640), 8 (16 & 800), 10 (13 & 800), 15 (11 & 800)	

22	*Gracilaria foliifera*	0 (22 & 100), 3 (20 & 100), 4 (18 & 100), 6 (16 & 100), 8 (15 & 135), 10 (14 & 135), 15 (13 & 135)	
23	*Gracilaria corticata*	0(16 & 100), 3 (14 & 100), 4 (13 & 100), 6 (12 & 100), 8 (11 & 110), 10 (10 & 110), 15 (9.5 & 110)	
24	*Gracilaria vermiculophylla*	7 (15.3 & 1064)	[30]
25	*Gracilaria blodgettii*	0 (32 & 750), 3 (26.2 & 500-750), 5 (22 & 500-750), 7 (24 & 5-750)	[34]
26	*Gracilaria cervicornis*	0 (39.3 & <50), 3 (26 & <50), 5 (24 & <50), 7 (22 & <50)	
27	*Gracilaria crassissima*	0 (30 & 180), 3 (22 & 800), 5 (16 & 1200), 7 (13.1 & 1390)	
28	*Gracilaria verrucosa*	0 (32 & 150), 1 (30 & 165), 5 (26.6 & 180), 10 (26 & 265), 15 (24 & 280)	[35]
29	*Gracilaria cornea*	0.5 (14.5 & 155), 1 (18.4 & 752), 3 (22.1 & 1647), 5 (19.4 & 1758)	[36]
30	*Pyropia yezoensis*	0 (26 & 160), 2 (24 & 400), 4 (23 & 820), 6 (20 & 1360), 8 (17 & 1150), 10 (14 & 1000)	[37]
31	*Pyropia haitanensis*	6 (10.45 & 180)	[38]
32	*Phycocalidia vietnamensis*	6 (10 & 157), 8 (8 & 350), 10 (7 & 597)	[10]
